# Longitudinal associations of rest-activity rhythms with executive function, plasma biomarkers, and clinical outcomes in older adults

**DOI:** 10.3389/fnagi.2026.1939060

**Published:** 2026-09-14

**Authors:** Daniel D. Callow, Nisha Rani, Adam P. Spira, Abhay Moghekar, Sarah Wanigatunga, Corinne Pettigrew, Anja Soldan, Marilyn Albert, Arnold Bakker

**Affiliations:** 1 Department of Psychiatry and Behavioral Sciences, Johns Hopkins University School of Medicine, Baltimore, MD, United States; 2 Department of Mental Health, Bloomberg School of Public Health, Baltimore, MD, United States; 3 Hopkins Center on Aging and Health, Baltimore, MD, United States; 4 Department of Neurology, Johns Hopkins School of Medicine, Baltimore, MD, United States

**Keywords:** actigraphy, Alzheimer’s disease, astrocytes, cognitive resilience, executive function, GFAP, longitudinal study, rest–activity rhythms

## Abstract

**Introduction:**

Daily patterns of activity, rest, and sleep, referred to as rest–activity rhythms (RARs), can be characterized by their day-to-day regularity, within-day fragmentation, and contrast between active and rest periods across the 24-h cycle. Disruptions (e.g., irregular schedules, fragmented activity, or reduced day-night contrast) have been associated with cognitive decline among older adults. Few studies have used repeated actigraphy to distinguish person-level average RARs from visit-specific deviations.

**Methods:**

The sample included 169 older adults without MCI or dementia at the first actigraphy visit, based on consensus clinical and neuropsychological review. Seven-day wrist actigraphy required at least three 24-h intervals with <5% missing data each; 122 participants had repeated assessments (362 observations total). RARs were characterized using three non-parametric features: (1) day-night contrast [relative amplitude (RA)], (2) day-to-day regularity [interdaily stability (IS)], and (3) within-day fragmentation [intradaily variability (IV)]. Each metric was decomposed into a person-level average and a visit-specific deviation from that average. Linear mixed-effects models examined associations with executive function and BMI-adjusted plasma markers of Alzheimer’s disease-related pathophysiology [pTau181/(Aβ42/Aβ40)], neuroaxonal injury (NfL), and astroglial activation (GFAP); exploratory Cox models evaluated incident MCI; and amyloid PET interaction models were conducted in participants with PET data (*n* = 141).

**Results:**

Higher average RA and IS and lower average IV were associated with higher executive function. At the visit level, higher-than-average RA and lower-than-average IV relative to each participant’s own average were associated with higher executive function at corresponding assessments. Higher average RA was associated with lower risk of incident MCI. RAR metrics were not significantly associated with GFAP, NfL, or pTau181/(Aβ42/Aβ40) in the plasma biomarker models. Amyloid PET status moderated within-person RAR associations with executive function: higher-than-usual RA and IS and lower-than-usual IV were more strongly associated with better executive function among amyloid PET-positive participants. For the plasma biomarkers, the between-person IS × amyloid PET interaction remained significant after correction only for pTau181/(Aβ42/Aβ40).

**Discussion:**

These findings highlight the value of repeated actigraphy for distinguishing average RAR levels from assessment-level departures from each participant’s own average RAR profile and suggest that visit-specific RAR variation may be particularly relevant to executive function among amyloid PET-positive older adults.

## Introduction

Rest–activity rhythms (RARs), reflecting changes in the 24-h organization of activity, rest, and sleep, have been associated with increased risk for cognitive decline (Duffy et al., 2015; Hood and Amir, 2017; Verma et al., 2023). In everyday terms, RARs capture how organized daily activity and rest patterns are, including the regularity of routines across days, the smoothness or fragmentation of activity within a day, and the contrast between periods of high and low activity. These rhythms are shaped jointly by endogenous circadian systems and both environmental factors (e.g., bright light) and habitual behaviors (e.g., meal timing, socialization), and can be measured unobtrusively in participants’ home environments using digital devices such as wrist actigraphy (Zhang et al., 2025). Because free-living activity profiles may not conform well to a simple cosine curve, non-parametric metrics are useful for directly quantifying day-to-day regularity, within-day fragmentation, and day–night contrast from actigraphy data (Xiao et al., 2021). Three commonly used features summarize key aspects of these rhythms: day-to-day regularity (interdaily stability [IS]; how consistent activity patterns are across days), within-day fragmentation (intradaily variability [IV]; how often individuals switch between activity and rest), and day–night contrast (relative amplitude [RA]; the degree to which active daytime periods are clearly separated from consolidated nighttime rest) (Xiao et al., 2021). These metrics were analyzed separately; throughout the manuscript, RAR organization is used as an overarching descriptive concept characterized by higher RA and IS and lower IV, not as a composite score.

Converging evidence from cross-sectional and prospective cohort studies of older adults indicates that less organized RARs, characterized behaviorally by reduced day–night separation (i.e., lower RA), lower day-to-day regularity (i.e., lower IS), and greater within-day fragmentation (i.e., higher IV), are associated with poorer cognitive performance, faster cognitive decline, and increased risk of incident cognitive impairment or dementia (Tranah et al., 2011; Lysen et al., 2020b; Sun et al., 2023; Haghayegh et al., 2024; Lu et al., 2024; Paget-Blanc et al., 2025). Prior studies have examined associations between RAR metrics and multiple cognitive domains, including memory, language, attention, processing speed, and executive function, but findings have varied across domains and populations (Kim et al., 2023, 2026; Zhang et al., 2025). Both Kim studies were cross-sectional and conducted in MCI or aMCI samples, whereas repeated-measures evidence for RAR metrics in older adults without MCI or dementia remains limited. Using a related within-/between-person approach, Shankar et al. (2026) reported concurrent associations between visit-specific physical activity and executive function; however, that study examined step count rather than RAR metrics. Prior cross-sectional BIOCARD studies, which included participants overlapping with the present sample, reported associations of actigraphy-derived rest and activity measures with executive function and brain measures (Callow et al., 2024a,2025b,a), motivating the present focus on executive function.

Beyond cognition, less organized RARs have been linked to neurobiological markers of Alzheimer’s disease and related dementias (ADRD) in cognitively unimpaired older adults, including amyloid PET burden, cortical and medial temporal neurodegeneration on MRI, and plasma markers of neurodegenerative disease (Lysen et al., 2020a; Roh et al., 2020; Spira et al., 2021; Liu et al., 2024; Van Egroo et al., 2024). However, much of this evidence is cross-sectional, and relatively few studies have examined repeated RAR assessments alongside repeated cognitive and plasma biomarker measures over multi-year follow-up in cognitively unimpaired older adults (Zhang et al., 2025). This limits understanding of whether person-level differences in average RAR levels, visit-specific departures from each participant’s average RAR profile, or both are associated with cognitive performance and biological markers relevant to ADRD. This distinction is important because a participant’s average RAR level across available visits addresses whether individuals who generally have more or less organized rhythms differ in cognition or biomarker levels, whereas visit-specific deviations address whether outcomes at a given actigraphy-linked assessment differ when an individual’s RAR is higher or lower than their own average (Curran and Bauer, 2011; Wang and Maxwell, 2015; Shankar et al., 2026).

Plasma biomarkers provide a minimally invasive and scalable approach to examining these relationships in cognitively unimpaired populations undergoing repeated assessments. Among commonly used markers, pTau181 and Aβ42/Aβ40 reflect AD-related tau phosphorylation and amyloid pathophysiology, respectively, whereas neurofilament light chain (NfL) indexes neuroaxonal injury and glial fibrillary acidic protein (GFAP) is commonly interpreted as a marker of astroglial activation. Prior population-based work has linked 24-h rest–activity patterns with plasma markers of neurodegenerative disease and AD-related pathophysiology, including NfL, GFAP, and plasma amyloid and tau markers (Lysen et al., 2020a; Van Egroo et al., 2024). However, longitudinal evidence using repeated RAR and concurrent plasma biomarker assessments remains limited. GFAP is of particular relevance in the context of RARs because astrocytes have been implicated in sleep–wake regulation, circadian signaling, metabolic support, and glymphatic clearance (Prolo et al., 2005; Keller et al., 2009; Xie et al., 2013; Fonken et al., 2014; Colwell and Ghiani, 2019; de Vivo and Bellesi, 2019; Nedergaard and Goldman, 2020; Li and Hu, 2025). RARs may relate to these markers through sleep–wake, vascular, metabolic, and inflammatory pathways; however, 7-day actigraphy and circulating biomarkers reflect different temporal windows, which may attenuate concurrent cross-modal associations.

To address these gaps, the present study leveraged repeated actigraphy, cognitive, and plasma biomarker data from the Biomarkers for Older Controls at Risk of Dementia (BIOCARD) study. A hybrid within-/between-person modeling framework was used to decompose each RAR metric into a between-person average across available actigraphy visits and a within-person visit-specific deviation from each participant’s own average (Curran and Bauer, 2011; Wang and Maxwell, 2015). This approach allowed evaluation of whether participants who generally had more organized RARs differed in executive function and biomarker levels, and whether assessment-level departures from a participant’s average RAR profile were associated with executive function or biomarker values measured at corresponding actigraphy-linked visits. These visit-specific deviations may reflect differences in behavioral rhythm organization during the actigraphy assessment period and, across multi-year follow-up, longer-term departures from an individual’s average RAR profile; however, they do not represent linear rates of change. Primary analyses examined whether person-level average RARs and visit-specific RAR deviations were associated with repeated executive function and plasma biomarker values measured at actigraphy-linked visits, with exploratory models examining associations between RAR metrics and risk of incident MCI. In addition, amyloid PET interaction models tested whether associations between RAR components and executive function or plasma biomarkers differed by amyloid PET status, motivated by the possibility that behavioral rhythm differences may be more apparent in the context of preclinical Alzheimer’s disease pathology (Sperling et al., 2014; Arenaza-Urquijo and Vemuri, 2018; Jack et al., 2024).

## Materials and methods

Data were obtained from the ongoing BIOCARD Study, a longitudinal investigation initiated in 1995 at the National Institutes of Health (NIH; Albert et al., 2014). At enrollment, participants were cognitively unimpaired, primarily middle-aged adults, approximately 75% of whom reported a family history of Alzheimer’s disease. Following a temporary study pause in 2005, the cohort was re-established in 2009 at the Johns Hopkins University (JHU). Participants underwent annual clinical and cognitive assessments throughout both study phases. During the NIH phase of the study, blood was collected approximately every other year. Starting in 2009, blood has been collected annually. Week long wrist-worn actigraphy recording began in 2015 and was conducted approximately every other year. The BIOCARD Study was approved by the Johns Hopkins Medicine Institutional Review Board and conducted in accordance with the Declaration of Helsinki. Written informed consent was obtained from all participants.

### Clinical and cognitive assessment

At each study visit, participants underwent comprehensive clinical evaluations, including a semi-structured interview based on the Clinical Dementia Rating (CDR) scale (Morris, 1993) and a battery of neuropsychological tests. The JHU BIOCARD Clinical Core determined annual consensus diagnoses, based on the NIA/AA Working Group criteria (Albert et al., 2011; McKhann et al., 2011). Participants were classified as cognitively unimpaired, MCI, impaired not MCI, or dementia following protocols consistent with the National Institute on Aging (NIA)/Alzheimer’s Disease Research Centers’ guidelines (Albert et al., 2011). The “impaired not MCI” classification reflects individuals with discrepancies between clinical and cognitive findings (e.g., cognitive concerns reported by the participant or informant that are not supported by neuropsychological testing, or declines in cognitive testing are noted, but there are no concerns by the participant or informant) and thus do not meet formal criteria for MCI. Consistent with prior BIOCARD studies (Callow et al., 2024a,2025c), participants classified as impaired not MCI at the first actigraphy visit were retained in the primary analytic sample because they did not meet formal criteria for MCI or dementia (*n* = 37/169). Sensitivity analyses excluding participants classified as impaired not MCI at baseline were conducted to confirm that the primary findings were not altered by inclusion of this subgroup. Participants who subsequently met criteria for MCI during follow-up were treated as converters in the incident MCI analyses.

Analyses included participants who completed at least one valid actigraphy assessment after actigraphy was added to BIOCARD and who did not meet criteria for MCI or dementia at the first valid actigraphy visit. Cognitive and plasma biomarker measures were linked to corresponding actigraphy assessments to create the actigraphy-linked repeated observations used in the primary models. The available analytic source file contained 458 actigraphy records from 246 participants, all of which had passed the recording-level validity criteria described below. One record lacked a linkable assessment date. After its removal, participant-level exclusions for unavailable diagnostic status (52 participants) or MCI or dementia at the first dated actigraphy record (25 participants) removed 95 dated records, yielding 362 eligible observations from 169 participants. Among these participants, 122 contributed repeated valid actigraphy assessments and 47 contributed one assessment.

Four cognitive domains were derived from a confirmatory factor analysis of 12 neuropsychological tests (Soldan et al., 2019). Executive function was selected *a priori* as the primary cognitive outcome. Executive function composites included the Wechsler Memory Scale Digit Span Backwards, Trail Making Test B, and the WAIS Digit Symbol Test.

### Actigraphy and rest–activity rhythm assessment

Approximately every other year, participants were asked to wear an Actiwatch-2 device (Philips Respironics, Bend, Oregon) continuously on the non-dominant wrist for seven consecutive days. Daily sleep logs documented times in and out of bed, naps, device removal, illness, and travel across time zones. We downloaded actigraphy data and inspected it using Actiware software (version 6.0.9; Philips Respironics). We excluded periods of non-wear, device malfunction, illness, procedures with anesthesia, or travel across time zones from analysis.

Consistent with prior work in this cohort (Callow et al., 2024a,b, 2025a,2025c), participants were required to contribute at least three 24-h intervals with <5% missing data each day to be included. Raw activity counts were recorded in 30-s epochs. Missing epoch-level values were imputed using the participant-specific mean activity at the corresponding time of day across valid days. Activity counts were log-transformed to reduce skewness (log[1 + counts per minute]) and aggregated into minute-level estimates, which we subsequently binned into 144 ten-minute intervals to construct daily rest–activity profiles. Daily curves were averaged across valid days to generate visit-level rest–activity rhythms (mean = 5.72 valid days per visit, standard deviation [SD] = 0.72). The number of valid days per visit was included as a covariate in all RAR analyses.

Three non-parametric RAR metrics were computed using the R (version 4.5.1) package ActCR (version 0.3.0): IS (day-to-day regularity), IV (fragmentation of the rhythm within a day), and RA (contrast between periods of highest and lowest activity within the 24-h cycle). We averaged participant-specific daily RAR metrics across valid days to obtain visit-level summaries.

### Plasma biomarker assessment

Plasma samples were assayed for Aβ40, Aβ42, pTau181, NfL, and GFAP using the NeuroToolKit panel with cobas Elecsys assays (Roche Diagnostics), as described previously (Soldan et al., 2024; Pettigrew et al., 2025). The current analyses focused on pTau181/(Aβ42/Aβ40), GFAP, and NfL. The Aβ42/Aβ40 ratio was used to account for individual differences in total Aβ production and reduce pre-analytic variability; lower values are generally interpreted as indicating greater amyloid pathophysiology. Higher pTau181 indicates greater Alzheimer’s disease-related tau pathophysiology. Accordingly, higher pTau181/(Aβ42/Aβ40) values indicate greater combined Alzheimer’s disease-related plasma biomarker burden. Recent work has directly evaluated this nested ratio and demonstrated strong discrimination of brain amyloidosis, supporting the combination of directionally complementary phosphorylated-tau and amyloid measures (Lehmann et al., 2025). The same formulation has also been used in prior BIOCARD work (Soldan et al., 2024). Plasma measures were natural log-transformed to reduce skewness and standardized using the distribution of participants’ first available biomarker measures. Values greater than 3 standard deviations above or below the transformed sample mean were defined as outliers and excluded from analyses. After outlier removal, biomarker values were re-standardized. Within the eligible actigraphy-linked dataset, application of this criterion excluded 10 observations from 8 participants for pTau181/(Aβ42/Aβ40), 1 observation from 1 participant for NfL, and 3 observations from 2 participants for GFAP. Outlier exclusions were biomarker-specific; an observation excluded for one biomarker remained eligible for analyses of the other biomarkers. Following outcome-specific outlier exclusion and complete-case filtering, the BMI-adjusted models included 334 observations for pTau181/(Aβ42/Aβ40), 343 observations for NfL, and 341 observations for GFAP. For the primary analytic dataset, plasma biomarker values were taken from blood draws aligned with the corresponding actigraphy-linked cognitive assessment.

### Vascular risk factors

At each actigraphy-linked visit, a time-varying vascular risk score was calculated from medical history and measured BMI. The score, which has been previously validated (Gottesman et al., 2017), is the sum of five binary vascular risk factors, where a value of 1 indicates the presence of the risk factor (recent/remote), and a value of 0 indicates its absence. These factors included hypertension, hypercholesterolemia, current smoking (within the last 30 days), obesity (i.e., measured body mass index [BMI] of 30 kg/m^2^ or higher), and diabetes. Data for vascular risk factors was obtained at the same visit as actigraphy. The vascular risk score was included as a model covariate, given that vascular risk can influence cognition and brain health (Kennedy and Raz, 2009). Because BMI can influence plasma biomarker concentrations, BMI was included as an additional covariate in plasma biomarker models (Yaffe et al., 2011). In these models, the obesity item was removed from the vascular risk score to avoid double-counting BMI.

### APOE genotype

APOE (apolipoprotein) genotyping was performed with digestion of polymerase chain reaction (PCR)-amplified genomic DNA utilizing restriction endonucleases by Athena Diagnostic in Worcester, Massachusetts. Individuals with at least one ε4 allele were categorized as ε4 carriers, and those without an ε4 allele were designated as non-carriers. The ε4 allele is the leading genetic risk factor for Alzheimer’s disease and has been shown to explain variance in, and modify associations between, modifiable risk factors, neuroimaging metrics, and cognition (Smith et al., 2011, 2016; Albert et al., 2014; Jeon et al., 2020). APOE ε4 carrier status was included as a covariate because it may explain variability in cognitive and biomarker outcomes.

### Amyloid PET image acquisition and processing

A subgroup of participants with actigraphy (*n* = 141) underwent amyloid PET imaging using carbon-11-labeled Pittsburgh compound B (^11^C-PiB). PET imaging was performed on a GE DISCOVERY RX PET/CT scanner in 3D acquisition mode. ^11^C-PiB scans were acquired approximately 40 min after intravenous administration of the tracer. Images were reconstructed using the 3D OP-OSEM algorithm with corrections for detector efficiency, decay, dead time, attenuation, and scatter. Standardized uptake value (SUV) images were generated by normalizing radioactivity concentration to injected dose and body weight and, employing a simplified reference tissue model with cerebellar gray matter serving as the reference region (Zhou et al., 2003).

Positron emission tomography preprocessing followed previously published BIOCARD procedures (Rani et al., 2023, 2025; Callow et al., 2025b,^2026^). Briefly, PET images were spatially smoothed with a 6 mm full-width-at-half-maximum Gaussian kernel, motion-corrected, averaged into a mean PET image, and registered to each participant’s T1-weighted MRI. Partial volume effects were corrected using a voxel-wise geometric transfer matrix approach implemented in PETSurfer. Standardized uptake value ratios (SUVRs) were calculated using cerebellar gray matter as the reference region, with regions of interest defined in each participant’s native MRI space. Global amyloid burden was quantified by averaging ^11^C-PiB SUVR across amyloid-associated cortical regions, including orbitofrontal, precuneus, anterior and posterior cingulate, parietal, temporal, and superior frontal regions. Participants were classified as amyloid positive using a mean cortical SUVR threshold of 1.40, consistent with prior literature and prior BIOCARD PET processing procedures (Callow et al., 2025b,c, 2026).

For amyloid PET interaction analyses, amyloid PET status was coded as a time-invariant ever-positive variable. Participants were classified as amyloid PET positive if they exceeded the positivity threshold at any available PET assessment and amyloid PET negative otherwise. This coding was used to identify participants with evidence of amyloid pathology during the broader follow-up period. PET was not obtained at every actigraphy-linked visit; therefore, a contemporaneous time-varying classification would have substantially reduced the analytic sample. The ever-positive definition may classify an earlier actigraphy visit according to PET positivity observed later, so these exploratory models indicate effect modification by evidence of amyloid pathology before or during follow-up rather than a contemporaneous PET-by-RAR effect.

### Statistical analysis

The primary analytic goal was to distinguish between-person differences in average RAR levels from within-person visit-specific deviations in RAR levels (Curran and Bauer, 2011; Wang and Maxwell, 2015). For each RAR metric, a hybrid within-/between-person modeling approach was used. For participant i at visit j, the observed RAR value was decomposed into two terms: the participant’s average RAR value across all available actigraphy visits (between-person component) and the visit-specific deviation (i.e., difference) from that participant’s own average (within-person component). For example, if a participant had RA values of 0.70, 0.75, and 0.80 across three visits, that participant’s average RA would be 0.75, and the within-person deviations would be −0.05, 0.00, and +0.05. Thus, the between-person term estimates whether participants who generally have higher or lower RAR values differ in outcomes, whereas the within-person term estimates whether outcomes differ at visits when a participant’s RAR value is higher or lower than that participant’s own average. Because actigraphy assessments were obtained approximately every other year, within-person RAR deviations were interpreted as assessment-level departures from each participant’s average RAR profile across study assessments, rather than day-to-day or week-to-week fluctuations. Because RARs and cognitive and fluid measures were obtained from the same assessments, the within-person coefficient represents a concurrent assessment-level association; it does not represent a linear RAR or cognitive slope, establish temporal ordering, or index overall RAR variability.

Linear mixed-effects models used the executive function composite measured at actigraphy-linked visits as the outcome. Separate models were run for RA, IV, and IS. Each model included both the between-person average and within-person deviation terms for the RAR metric as predictors, along with baseline age, sex, education, APOE ε4 carrier status, vascular risk score, time since baseline, and valid actigraphy days. Random intercepts for participant accounted for within-person correlation across repeated assessments. A parallel set of linear mixed-effects models examined associations between RAR components and visit-specific plasma biomarker values, including pTau181/(Aβ42/Aβ40), NfL, and GFAP. Separate models were run for each plasma biomarker and each RAR metric. The plasma models additionally included BMI to account for its known influence on plasma biomarker concentrations. This hybrid approach was selected to use all available actigraphy-linked observations while distinguishing person-average RAR levels from same-visit departures from each participant’s own average RAR profile; the models were not designed to estimate coupled rates of change in RARs and cognitive or plasma biomarker outcomes. All continuous predictors were standardized to facilitate comparison of effect sizes. Model estimates are reported as standardized beta coefficients with standard errors and *p*-values.

Exploratory Cox proportional hazards models evaluated associations between RAR components and incident MCI. Time-to-event was defined from the first valid actigraphy assessment to incident MCI diagnosis or censoring at the last available clinical diagnosis. Separate Cox models were run for RA, IV, and IS, with both between-person and within-person RAR terms included in each model. Models were adjusted for baseline age, sex, education, APOE ε4 carrier status, vascular risk score, and valid actigraphy days. These models were considered exploratory because of the small number of incident MCI events.

To evaluate whether associations differed by amyloid PET status, exploratory interaction models were conducted in the subset of participants with available amyloid PET data. These models included interaction terms between amyloid PET status and both the between-person and within-person components of each RAR metric. Covariates were the same as the primary models, described above. Amyloid PET-negative participants served as the reference group. Significant interaction terms were interpreted as evidence that the association between a RAR component and the outcome differed among amyloid PET-positive participants relative to amyloid PET-negative participants. For selected nominally significant interactions, model-based simple slopes and 95% confidence intervals were estimated for PET-negative and PET-positive participants from the fitted interaction models. Separate stratified models were used only to generate the visualizations in Figure 1 and were not used for statistical inference.

**FIGURE 1:**
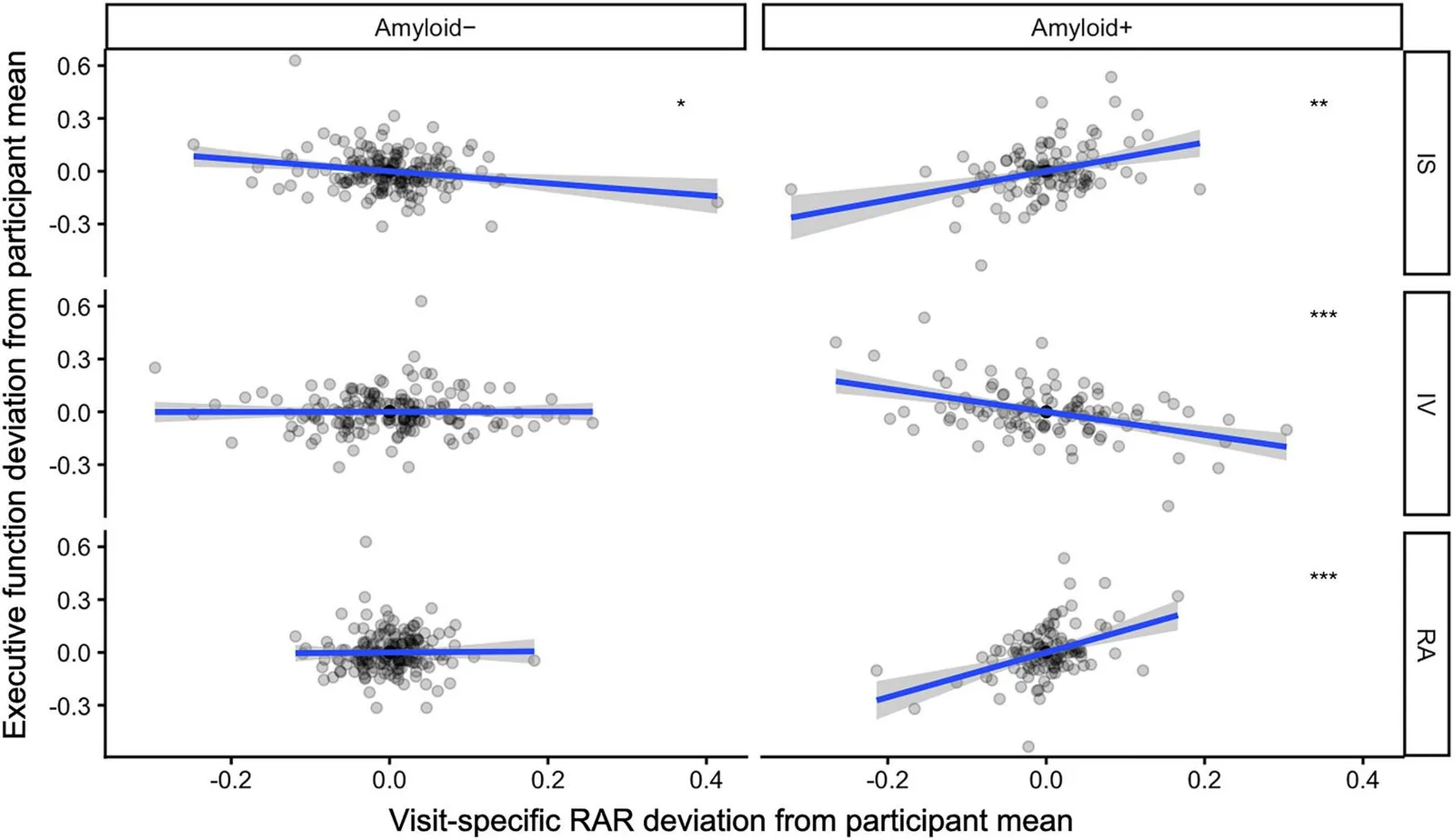
Visualization of within-person associations between rest–activity rhythm deviations and executive function by amyloid PET status. Panels show visit-specific deviations from each participant’s own mean for interdaily stability (IS), intradaily variability (IV), and relative amplitude (RA) among amyloid PET-negative (Amyloid–; *n* = 87) and amyloid PET-positive (Amyloid+; *n* = 54) participants. Positive RAR deviation values indicate visits when a participant’s RAR value was higher than that participant’s average across available actigraphy visits; negative values indicate visits when the RAR value was lower than that participant’s average. Executive function deviations are shown for visualization of within-person patterns only; the primary linear mixed-effects models used the visit-specific executive function composite as the outcome, included participant random intercepts, and adjusted for time since baseline and other covariates. Deviations represent visit-specific departures from each participant’s own average and do not necessarily indicate linear change over time. Lines show fitted associations within amyloid PET groups. *p < 0.05; **p < 0.01; ***p < 0.001.

Sensitivity analyses assessed robustness of the main executive-function findings by (a) restricting analyses to participants with repeated actigraphy assessments and (b) restricting analyses to actigraphy-linked observations with at least five valid actigraphy days and (c) by excluding participants classified as impaired not MCI at the first actigraphy visit.

All analyses were conducted in R version 4.5.1 using the lme4, lmerTest, and survival packages. Two-sided nominal *p*-values less than 0.05 were considered statistically significant for the primary executive function, plasma biomarker, and exploratory Cox proportional hazards models. Because the amyloid PET interaction analyses were exploratory and included multiple related tests, multiplicity checks were additionally conducted for these models. For executive function interaction models, *p*-values were adjusted across six interaction terms, reflecting between-person and within-person amyloid PET interaction terms for RA, IV, and IS. For plasma biomarker interaction models, *p*-values were adjusted across 18 interaction terms, reflecting between-person and within-person amyloid PET interaction terms for three RAR metrics across three plasma biomarkers. Bonferroni-adjusted and false discovery rate-adjusted *p*-values were calculated to distinguish robust interaction findings from nominal exploratory associations. Nominal *p*-values are reported in the main tables, with corrected results summarized in the Results.

## Results

### Participant characteristics

Baseline characteristics of the 169 participants included in the primary analytic sample are summarized in Table 1. Participants contributed 362 total actigraphy-linked observations, with an average of 2.14 actigraphy visits per participant (SD = 0.91). Forty-seven participants contributed one valid actigraphy visit, 64 contributed two valid actigraphy visits, and 58 contributed three or more valid actigraphy visits. Mean follow-up duration across the full sample was 3.18 years (SD = 2.63); among participants with repeated actigraphy assessments, mean follow-up was 4.40 years (SD = 2.04; range = 1.80–7.65 years).

**TABLE 1:** Participant baseline characteristics.

Baseline participant characteristics	Full sample (*n* = 169)	Non-converters (*n* = 157)	MCI converters (*n* = 12)
Female sex, *N* (%)	113 (66.9%)	104 (66.2%)	9 (75.0%)
Age	71.1 (8.7)	71.0 (8.9)	71.7 (5.9)
Years of education	17.2 (2.3)	17.2 (2.3)	17.2 (2.5)
APOE*-*ε4 carriers, *N* (%)	54 (32.0%)	48 (30.6%)	6 (50.0%)
Vascular risk score	1.49 (1.15)	1.55 (1.14)	0.83 (1.03)
Relative amplitude	0.782 (0.09)	0.785 (0.08)	0.747 (0.10)
Intradaily variability	0.497 (0.17)	0.497 (0.18)	0.493 (0.08)
Interdaily stability	0.792 (0.09)	0.792 (0.10)	0.794 (0.08)
Executive function composite	0.129 (0.44)	0.159 (0.44)	−0.262 (0.44)
Valid actigraphy days	5.60 (0.83)	5.59 (0.85)	5.67 (0.65)
BMI, kg/m^2^	26.92 (5.13)	–	–
Actigraphy-linked analytic data availability			
Valid actigraphy observations, total	362	–	–
Actigraphy visits per participant	2.14 (0.91)	–	–
Actigraphy follow-up among participants with repeated actigraphy, years	4.40 (2.04), range 1.80–7.65	–	–
Actigraphy follow-up, years	3.18 (2.6)	–	–
Executive function LME analytic observations, observations / participants	344 / 166	–	–
Plasma biomarker LME analytic observations	pTau181/(Aβ42/Aβ40): 334; NfL: 343; GFAP: 341	–	–
Participants with 1 valid actigraphy visit, *N* (%)	47 (27.8%)	–	–
Participants with 2 valid actigraphy visits, *N* (%)	64 (37.9%)	–	–
Participants with ≥3 valid actigraphy visits, *N* (%)	58 (34.3%)	–	–
Amyloid PET available, *N* (%)	141 (83.4%)	–	–
Amyloid PET positive among PET subset, *N* (%)	54 (38.3%)	–	–

Values reflect mean (SD) unless otherwise indicated. Baseline characteristics were taken from the first valid actigraphy-linked assessment. Actigraphy follow-up was calculated from the first to last valid actigraphy assessment; participants with only one valid actigraphy assessment contributed 0 years to the full-sample follow-up estimate. Executive function and plasma biomarker analytic observations reflect visit-specific outcome values available for the primary linear mixed-effects models at visits aligned with valid actigraphy assessments; these counts do not represent the full BIOCARD cognitive, clinical, or plasma biomarker follow-up. Amyloid PET status was coded as ever positive across available PET assessments. APOE, apolipoprotein E; BMI, body mass index; LME, linear mixed-effects; MCI, mild cognitive impairment; PET, positron emission tomography.

Participants were predominantly female (66.9%), with a mean baseline age of 71.1 years (SD = 8.7), mean education of 17.2 years (SD = 2.3), and 32.0% were APOE ε4 carriers. Over follow-up, 12 participants progressed to MCI, with a mean time to MCI diagnosis of 2.55 years from the first valid actigraphy assessment (range = 1.75–6.23 years).

### Rest–activity rhythm metrics and executive function

Results of the linear mixed-effects models examining associations between RAR metrics and executive function are shown in Table 2. For the between-person RAR components, participants with higher person-average RA across available actigraphy visits had higher executive function scores (β = 0.103, SE = 0.030, *p* = 0.001). Higher person-average IS was also associated with higher executive function scores (β = 0.079, SE = 0.031, *p* = 0.012), whereas higher person-average IV was associated with lower executive function scores (β = −0.067, SE = 0.031, *p* = 0.032).

**TABLE 2:** Linear mixed-effects models assessing associations between non-parametric rest–activity rhythm metrics and executive function.

Predictor	Beta estimate	Standard error (SE)	*P*-value
Relative amplitude (RA), between-person mean	0.103	0.030	0.001**
Relative amplitude (RA), within-person deviation	0.023	0.008	0.004**
Intradaily variability (IV), between-person mean	−0.067	0.031	0.032*
Intradaily variability (IV), within-person deviation	−0.019	0.008	0.025*
Interdaily stability (IS), between-person mean	0.079	0.031	0.012*
Interdaily stability (IS), within-person deviation	0.007	0.008	0.369

Separate models were run for RA, IV, and IS. Each model included the between-person mean and within-person deviation component of the RAR metric. Models were adjusted for education, APOE ε4 status, sex, vascular risk score, baseline age, time since baseline, and valid actigraphy days.

*p < 0.05; **p < 0.01. β-values are standardized regression coefficients.

For the within-person component, visits when participants had higher RAs relative to their own average were associated with higher executive function scores at that same visit (β = 0.023, SE = 0.008, *p* = 0.004). Visits when participants had higher IV relative to their own average were associated with lower executive function scores (β = −0.019, SE = 0.008, *p* = 0.025). Within-person deviations in IS were not significantly associated with executive function (β = 0.007, SE = 0.008, *p* = 0.369).

### Rest–activity rhythm metrics and incident MCI

Results of the exploratory Cox proportional hazards models examining incident MCI are shown in Table 3. Higher between-person average RA was associated with lower risk of incident MCI (HR = 0.499, 95% CI: 0.311–0.801, *p* = 0.004). Within-person deviations in RA were not associated with incident MCI risk (HR = 0.918, 95% CI: 0.685–1.230, *p* = 0.565). Neither between-person nor within-person components of IV or IS were significantly associated with incident MCI. These findings should be interpreted cautiously given the small number of incident MCI events.

**TABLE 3:** Exploratory Cox proportional hazards models examining associations between non-parametric rest–activity rhythm metrics and risk of incident MCI.

Predictor	Hazard ratio (HR)	95% confidence interval (CI)	*P*-value
Relative amplitude (RA), between-person mean	0.499	0.311–0.801	0.004**
Relative amplitude (RA), within-person deviation	0.918	0.685–1.230	0.565
Intradaily variability (IV), between-person mean	1.654	0.804–3.403	0.172
Intradaily variability (IV), within-person deviation	0.988	0.667–1.462	0.950
Interdaily stability (IS), between-person mean	0.868	0.475–1.589	0.647
Interdaily stability (IS), within-person deviation	0.846	0.594–1.206	0.356

Separate Cox proportional hazards models were run for RA, IV, and IS. Each model included the between-person mean and within-person deviation component of the RAR metric as simultaneous model predictors. Models were adjusted for education, APOE ε4 status, sex, vascular risk score, baseline age, and valid actigraphy days.

**p < 0.01.

### Rest–activity rhythm metrics and plasma biomarkers

Results of BMI-adjusted plasma biomarker models are shown in Table 4. No significant associations were observed between the between-person average or within-person deviation components of RA, IV, or IS and pTau181/(Aβ42/Aβ40), NfL, or GFAP. For pTau181/(Aβ42/Aβ40), all RAR associations were nonsignificant (all *p* ≥ 0.419). For NfL, neither between-person nor within-person RAR components were significant (all *p* ≥ 0.219). For GFAP, no between-person or within-person RAR component was statistically significant (all *p* ≥ 0.194).

**TABLE 4:** BMI-adjusted linear mixed-effects models assessing associations between non-parametric rest–activity rhythm metrics and plasma biomarkers.

Plasma biomarker	RAR metric	Between-person mean β	*P*-value	Within-person deviation β	*P*-value
pTau181/(Aβ42/Aβ40)	Relative amplitude (RA)	−0.022	0.789	0.003	0.901
pTau181/(Aβ42/Aβ40)	Intradaily variability (IV)	0.066	0.419	−0.005	0.824
pTau181/(Aβ42/Aβ40)	Interdaily stability (IS)	0.026	0.754	0.012	0.543
NfL	Relative amplitude (RA)	−0.065	0.308	0.026	0.283
NfL	Intradaily variability (IV)	0.079	0.219	0.004	0.872
NfL	Interdaily stability (IS)	−0.074	0.241	0.009	0.707
GFAP	Relative amplitude (RA)	0.079	0.194	−0.001	0.941
GFAP	Intradaily variability (IV)	0.019	0.762	−0.016	0.401
GFAP	Interdaily stability (IS)	0.061	0.316	0.015	0.419

Separate linear mixed-effects models were run for each plasma biomarker and RAR metric. Each model included the between-person mean and within-person deviation component of the RAR metric. Models were adjusted for education, APOE ε4 status, sex, vascular risk score, baseline age, time since baseline, valid actigraphy days, and BMI. β-values are standardized regression coefficients. Analytic observations: pTau181/(Aβ42/Aβ40), 334; NfL, 343; GFAP, 341.

### Amyloid PET interaction models

Amyloid PET interaction models were conducted among participants with available amyloid PET data. Of the 141 participants with amyloid PET data, 54 (38%) were classified as amyloid PET positive and 87 (62%) were classified as amyloid PET negative. Interaction models formally tested whether associations between RAR components and outcomes differed by amyloid PET status (see Figure 1 and Table 5). Because amyloid PET status was defined using the time-invariant ever-positive approach, these exploratory interactions reflect effect modification by evidence of amyloid pathology before or during follow-up rather than a contemporaneous PET-by-RAR effect.

**TABLE 5:** Amyloid PET × rest–activity rhythm interaction models for executive function and plasma biomarkers.

Outcome	RAR metric	Between-person mean × amyloid PET β	*P*-value	Within-person deviation × amyloid PET β	*P*-value
Executive function	RA	0.024	0.742	0.049	0.003**
	IV	−0.060	0.409	−0.057	0.001**
	IS	0.037	0.619	0.068	<0.001***
GFAP (BMI-adjusted)	RA	0.175	0.131	−0.065	0.086
	IV	−0.138	0.242	0.064	0.107
	IS	0.312	0.008**	−0.013	0.737
NfL (BMI-adjusted)	RA	0.175	0.192	−0.048	0.327
	IV	−0.154	0.247	0.090	0.082
	IS	0.088	0.519	−0.043	0.409
pTau181/(Aβ42/Aβ40) (BMI-adjusted)	RA	−0.080	0.633	0.028	0.496
	IV	−0.333	0.041*	−0.001	0.991
	IS	0.555	<0.001***	0.058	0.175

Separate interaction models were run for each outcome and RAR metric. Models tested interactions between amyloid PET status, and both the between-person mean and within-person deviation components of each RAR metric. Executive function models were adjusted for education, APOE ε4 status, sex, vascular risk score, baseline age, time since baseline, and valid actigraphy days. Plasma biomarker models were additionally adjusted for BMI. Amyloid PET-negative participants served as the reference group. Nominal *p*-values are shown in the table. Bonferroni- and FDR-adjusted *p*-values were calculated within outcome families for exploratory interaction models. For executive function, all three within-person RAR × amyloid PET interactions remained significant after correction. For plasma biomarkers, only the between-person IS × amyloid PET interaction for pTau181/(Aβ42/Aβ40) remained significant after Bonferroni and FDR correction across the 18 plasma interaction tests; the analogous GFAP interaction was nominally significant but did not survive correction. Complete-case models included 301 observations from 140 participants for executive function, 298 observations from 139 participants for GFAP, 300 observations from 140 participants for NfL, and 293 observations from 138 participants for pTau181/(Aβ42/Aβ40).

*p < 0.05; **p < 0.01; ***p < 0.001.

For executive function, amyloid PET status significantly moderated within-person RAR associations, but not between-person RAR associations. Specifically, the within-person RA × amyloid PET interaction was significant (β = 0.049, SE = 0.016, nominal *p* = 0.003; Bonferroni-adjusted *p* = 0.017; FDR-adjusted *p* = 0.006), indicating that visits with RA values above a participant’s own average were more strongly associated with higher executive function among amyloid PET-positive participants than among amyloid PET-negative participants. The within-person IV × amyloid PET interaction was also significant (β = −0.057, SE = 0.017, nominal *p* = 0.001; Bonferroni-adjusted *p* = 0.006; FDR-adjusted *p* = 0.003), indicating that visits with IV values above a participant’s own average were more strongly associated with lower executive function among amyloid PET-positive participants. Finally, the within-person IS × amyloid PET interaction was significant (β = 0.068, SE = 0.017, nominal *p* < 0.001; Bonferroni-adjusted *p* < 0.001; FDR-adjusted *p* < 0.001), indicating that visits with IS values above a participant’s own average were more strongly associated with higher executive function among amyloid PET-positive participants. Between-person RAR × amyloid PET interactions were not significant for RA, IV, or IS.

For plasma biomarkers, nominal interaction findings were concentrated in the between-person components. For GFAP, the between-person IS × amyloid PET interaction was nominally significant (β = 0.312, SE = 0.117, nominal *p* = 0.008), but did not remain significant after correction across the 18 plasma interaction tests (Bonferroni-adjusted *p* = 0.149; FDR-adjusted *p* = 0.075). Model-based simple slopes indicated that higher between-person IS was associated with higher GFAP among amyloid PET-positive participants (β = 0.263, 95% CI 0.084–0.442, *p* = 0.004), but not among amyloid PET-negative participants (β = −0.049, 95% CI −0.198 to 0.100, *p* = 0.514). No other GFAP interaction reached nominal significance. For pTau181/(Aβ42/Aβ40), the between-person IV × amyloid PET interaction was nominally significant (β = −0.333, SE = 0.162, nominal *p* = 0.041), but did not survive correction (Bonferroni-adjusted *p* = 0.745; FDR-adjusted *p* = 0.248). The between-person IS × amyloid PET interaction was significant (β = 0.555, SE = 0.161, nominal *p* < 0.001) and remained significant after both Bonferroni and FDR correction (both adjusted *p* = 0.013). Higher between-person IS was associated with higher pTau181/(Aβ42/Aβ40) among amyloid PET-positive participants (β = 0.423, 95% CI 0.179–0.667, *p* < 0.001), but not among amyloid PET-negative participants (β = −0.132, 95% CI −0.339 to 0.076, *p* = 0.212). No within-person pTau181/(Aβ42/Aβ40) interactions were significant. No amyloid PET interactions involving NfL reached nominal significance.

### Sensitivity analyses

Sensitivity analyses supported the robustness of the primary executive function findings. When analyses were restricted to participants with repeated actigraphy assessments, between-person RA and within-person RA remained significantly associated with executive function, and within-person IV remained significantly associated with executive function. Findings were also consistent when analyses were restricted to visits with at least five valid actigraphy days (*n* = 158 participants) and when participants classified as impaired not MCI at the first actigraphy visit were excluded (*n* = 37 participants).

## Discussion

The primary goal of this study was to evaluate whether person-level average RARs and visit-specific deviations in RARs were associated with executive function, plasma biomarkers, and subsequent clinical status among older adults without MCI or dementia at the first valid actigraphy visit who underwent repeated actigraphy, cognitive, and plasma biomarker assessments. This analysis was motivated by the need to better understand how everyday behavioral patterns relate to cognitive performance and biological markers relevant to Alzheimer’s disease and related dementias, and whether these relationships differ in the presence of underlying AD pathology.

The findings suggested that participants with more organized average daily behavioral patterns, characterized by higher contrast between periods of activity and rest [greater relative amplitude (RA)], greater day-to-day regularity [interdaily stability (IS)], and less fragmentation of activity within the day [lower intradaily variability (IV)], demonstrated better executive function. In addition, within individuals, visits with RA values above each participant’s own average and IV values below each participant’s own average were associated with better executive function at corresponding assessments. Exploratory analyses showed that higher average RA was associated with lower risk of incident mild cognitive impairment; however, given the small number of progressors, these analyses should be interpreted with caution. In the plasma biomarker models, RAR components were not significantly associated with GFAP, NfL, or pTau181/(Aβ42/Aβ40) levels. Amyloid PET interaction models indicated that within-person RAR associations with executive function were stronger among amyloid PET positive compared to amyloid PET-negative individuals. Plasma biomarker interaction findings were more limited: the between-person IS × amyloid PET interaction remained significant after correction for pTau181/(Aβ42/Aβ40), whereas the analogous GFAP interaction was nominally significant but did not survive correction.

By decomposing RAR metrics into person-level averages and visit-specific deviations, this study extends prior work by distinguishing two related but distinct sources of variability in daily rhythm organization (Curran and Bauer, 2011; Wang and Maxwell, 2015; Du et al., 2024). The person-level average term addresses whether participants who generally have higher or lower RAR values across available actigraphy visits differ in executive function or biomarker levels. The within-person deviation term addresses whether outcomes differ at visits when a participant’s RAR value is higher or lower than that participant’s own average. This distinction is important because the within-person term does not represent a linear rate of change over time or a participant-level measure of overall RAR variability; rather, it captures assessment-level departures from an individual’s own average rhythm profile. The findings suggest that both person-level average RAR levels and visit-specific deviations provide relevant information about executive function, while avoiding the assumption that a single baseline actigraphy assessment fully represents an individual’s rhythm organization over follow-up. The standardized associations with executive function were small: significant between-person estimates ranged from β = 0.067–0.103, and significant within-person estimates ranged from β = 0.019–0.023. No established threshold indicates that differences of this magnitude are clinically meaningful for an individual; at present, they are most informative at the population level. The study was designed to characterize repeated-assessment associations and distinguish between- and within-person sources of variation, not to derive individual-level clinical cut points or establish predictive utility. Accordingly, a within-person coefficient should be interpreted as concurrent covariation around an individual’s usual RAR level at linked assessments, not as evidence that a change in RAR preceded or caused a change in cognition or biomarkers.

These findings build directly on insights summarized by Zhang et al. (2025), who reviewed 26 studies examining links between RARs and cognition, including 19 cross-sectional and 7 longitudinal studies (Zhang et al., 2025). In that review, most longitudinal studies used a single baseline RAR assessment to predict later cognitive outcomes. By incorporating repeated RAR measurements, the present study addresses this limitation and suggests that both average RAR levels and visit-specific deviations in RARs are associated with executive function. Consistent with findings by Zhang et al. (2025) and prior cross-sectional findings in BIOCARD (Callow et al., 2024b,2025a), executive function was associated with RAR organization. Zhang et al. (2025) also highlighted several potential candidate mechanistic pathways linking RARs to cognition, including circadian regulation, sleep–wake homeostasis, and neuroinflammatory activity, which provide a conceptual framework for interpreting these findings. Because the study is observational, these associations may be bidirectional: RAR organization could covary with executive function through sleep–wake regularity, activity timing, or circadian alignment; conversely, better executive function may facilitate more structured routines. Neurodegeneration, vascular health, mood, mobility, or social schedules may also influence both RAR organization and cognition.

A key observation in the present study is that amyloid PET status moderated within-person RAR associations with executive function. Among amyloid PET-positive participants, visits with RA and IS values above each participant’s own average and IV values below each participant’s own average were more strongly associated with better executive function. However, because the ever-positive definition may classify an earlier actigraphy-linked assessment according to PET positivity identified later, temporal ordering between amyloid status and the within-person RAR associations cannot be established. These interactions therefore should not be interpreted as contemporaneous effects of amyloid pathology. Within these temporal limitations, this pattern suggests that visit-specific RAR deviations may be particularly relevant to executive function among participants with evidence of amyloid pathology during the broader follow-up period. RAR metrics partly reflect the timing, regularity, and consolidation of physical activity and rest/sleep patterns, and similar patterns have been observed in prior work, where higher levels of physical activity were more strongly associated with cognitive performance among individuals with elevated amyloid burden but showed weaker or null associations in those without significant amyloid pathology (Dougherty et al., 2025; Yau et al., 2025; Zhu et al., 2025). Although sleep characteristics are also associated with both amyloid burden and cognitive function (Osorio et al., 2013; Sprecher et al., 2017; Insel et al., 2021; Dzierzewski et al., 2022; Owusu et al., 2023), their role as effect modifiers of cognition–pathology relationships have been less extensively explored. In this context, the present findings suggest that daily behavioral rhythm organization may be an informative behavioral correlate of cognitive performance among amyloid PET-positive older adults. However, these results should be interpreted as evidence of effect modification rather than evidence of cognitive resilience. Demonstrating resilience would require showing that RAR organization attenuates the association between Alzheimer’s disease pathology and cognitive impairment or decline (Memel et al., 2021; Stern et al., 2023; Svenningsson et al., 2024). Thus, the present findings are consistent with, but do not establish, a resilience-based framework.

Exploratory analyses further showed that higher average RA was associated with lower risk of incident MCI, independent of demographic, genetic, and vascular factors. Although the number of conversion events was small, this finding is consistent with prior cohort studies demonstrating that weaker or more fragmented rest–activity rhythms are associated with incident cognitive impairment and dementia (Tranah et al., 2011; Haghayegh et al., 2024). However, with only 12 events relative to the number of adjusted covariates, the estimate is vulnerable to overfitting and model instability and should be considered hypothesis-generating. Replication in studies with more incident MCI cases is needed.

In contrast to the executive function findings, the plasma biomarker models did not show significant associations between RAR components and GFAP, NfL, or pTau181/(Aβ42/Aβ40). Thus, the present results do not support broad associations between RAR organization and circulating markers of astroglial activation, neuroaxonal injury, or AD-related plasma biomarker burden in the full sample after accounting for BMI. Prior population-based work has produced mixed findings. In the Rotterdam Study, sleep and 24-h activity rhythm disturbances were not consistently associated with plasma NfL, Aβ40, Aβ42, or total tau after adjustment and correction for multiple testing (Lysen et al., 2020a). By contrast, Van Egroo et al. (2024) reported that greater rest–activity rhythm fragmentation was associated with higher plasma NfL and GFAP in cognitively unimpaired adults, with evidence that several RAR–plasma biomarker associations were stronger in males. Several study-specific factors may have contributed to these null results, including the modest biomarker sample, measurement variability, and the different timescales captured by a 7-day actigraphy assessment versus circulating biomarkers. RAR–cognition associations may also reflect behavioral, vascular, or sleep–wake processes not captured by the assayed plasma markers. The present findings are therefore more consistent with studies showing limited or inconsistent plasma biomarker associations, while also underscoring that RAR–biomarker relationships may depend on sample characteristics, sex, biomarker platform, RAR metric, amyloid burden, and covariate adjustment.

Amyloid PET interaction analyses for plasma biomarkers were more limited than the executive-function interactions. The between-person IS × amyloid PET interaction for pTau181/(Aβ42/Aβ40) remained significant after correction, with a positive association among amyloid PET-positive participants and no significant association among amyloid PET-negative participants. The analogous GFAP interaction showed the same directional pattern in the group-specific estimates but did not survive correction for the 18 plasma interaction tests. These exploratory findings did not extend consistently across RAR metrics or biomarkers and should not be interpreted as a general plasma-biomarker pattern. Therefore, it is not possible to determine whether these interaction findings reflect rhythm-related biological processes, amyloid-related differences, or shared upstream mechanisms. GFAP is an intermediate filament protein expressed in astrocytes, which play central roles in sleep–wake regulation, circadian signaling, metabolic support, and glymphatic clearance (Keller et al., 2009; Xie et al., 2013; de Vivo and Bellesi, 2019; Nedergaard and Goldman, 2020; Li and Hu, 2025). At the same time, plasma GFAP may be influenced by peripheral sources and clearance dynamics, and its interpretation in cognitively unimpaired populations remains an active area of investigation (Youn et al., 2025; Zhu et al., 2025). Future studies with larger amyloid PET samples, longitudinal PET measures, and repeated plasma biomarkers are needed to clarify these relationships.

Several limitations should be considered. The sample was relatively small, highly educated, demographically homogeneous, and enriched for family history of Alzheimer’s disease (approximately 75% at enrollment), which may limit generalizability to more socioeconomically, educationally, and racially and ethnically diverse populations and to routine clinical settings. These cohort characteristics may restrict variability in cognitive performance and daily routines and could also influence amyloid prevalence and effect-modification estimates. Replication is needed in community-based cohorts with broader educational, socioeconomic, racial and ethnic, and health and functional profiles, including samples drawn from routine clinical settings. Although repeated actigraphy is a strength, the average number of actigraphy visits per participant was modest, and 47 participants contributed only one valid actigraphy assessment. Participants with one actigraphy visit contributed to between-person estimates but provided no within-person variation for estimating within-person associations. The present analyses also did not estimate coupled rates of change between RAR metrics and executive function or plasma biomarkers, nor did they directly test whether participants with greater overall RAR variability had better or worse outcomes. Because repeated actigraphy assessments spanned several years, visit-specific deviations may reflect both assessment-period differences in behavior and longer-term departures from an individual’s average RAR profile; adjustment for time since baseline accounts for average temporal trends but cannot fully separate these sources. The number of participants who converted to MCI was small, reducing statistical power for clinical progression analyses. Actigraphy provides an ecologically valid measure of behavioral rhythms but cannot distinguish endogenous circadian processes from behavioral influences. Because light exposure was not analyzed, we cannot determine whether the RAR–cognition associations reflect differences in circadian entrainment by light versus behavioral scheduling or other influences. Amyloid PET status was coded as an ever-positive variable across available PET assessments, which allowed classification of participants with evidence of amyloid pathology during the broader study period but did not require PET to be contemporaneous with each actigraphy visit. As an observational study, causal inference is not possible, and bidirectional relationships between cognition, health status, and daily rhythm organization are likely. Future studies incorporating multimodal assessments, including neuroimaging and physiological measures of circadian regulation, will be important for clarifying underlying mechanisms and temporal relationships.

In summary, more organized average RARs were associated with better executive function among older adults without MCI or dementia at the first valid actigraphy visit, and visit-specific deviations toward clearer day–night contrast and lower within-day fragmentation were associated with better executive function at corresponding actigraphy-linked assessments. Higher average RA was also associated with lower risk of incident MCI in exploratory analyses. Amyloid PET interaction findings suggest that visit-specific RAR deviations may be particularly relevant to executive function among amyloid PET-positive participants. In contrast, primary BMI-adjusted plasma biomarker models were null. Exploratory interaction analyses provided multiplicity-corrected evidence that amyloid PET status moderated the between-person IS association with pTau181/(Aβ42/Aβ40), whereas the analogous GFAP interaction was nominally significant but did not survive correction. In future studies, repeated actigraphy could establish a person-specific behavioral baseline and identify sustained departures for evaluation alongside cognitive and biological measures. In a research setting, departures that exceed expected measurement variability could be evaluated alongside cognitive change and established risk markers or multimodal risk models. Before clinical use, such approaches would need to demonstrate measurement reliability, incremental predictive value beyond demographic, genetic, and health information, clinically meaningful thresholds, actionability, and performance in diverse populations. The present findings do not support immediate clinical monitoring or risk thresholds; these applications require prospective validation and replication.

## Data Availability

Data used in these analyses are available through standard application procedures described on the BIOCARD website (www.biocard-se.org). Additional information or materials relating to the analysis are available from the corresponding author upon reasonable request.
